# Living at Higher Altitude and Incidence of Overweight/Obesity: Prospective Analysis of the SUN Cohort

**DOI:** 10.1371/journal.pone.0164483

**Published:** 2016-11-03

**Authors:** Jesús Díaz-Gutiérrez, Miguel Ángel Martínez-González, Juan José Pons Izquierdo, Pedro González-Muniesa, J. Alfredo Martínez, Maira Bes-Rastrollo

**Affiliations:** 1 University of Navarra, Department of Preventive Medicine and Public Health, School of Medicine, Pamplona, Spain; 2 IDISNA Navarra’s Health Research Institute, Pamplona, Spain; 3 CIBER Fisiopatología de la Obesidad y Nutrición (CIBERobn), Instituto de Salud Carlos III, Madrid, Spain; 4 Harvard TH Chan School of Public Health, Boston, United States of America; 5 University of Navarra, Department of History, Art History, and Geography, School of Humanities and Social Sciences, Pamplona, Spain; 6 University of Navarra, Department of Nutrition and Food Sciences and Physiology, School of Pharmacy, Pamplona, Spain; Hunter College, UNITED STATES

## Abstract

**Background:**

Residence at high altitude has been associated with lower obesity rates probably due to hypoxia conditions. However, there is no evidence of this association in a free-living population.

**Objectives:**

We assessed the association between the altitude where each participant of a Spanish cohort (the SUN Project) was living and the incidence of overweight/obesity.

**Methods:**

The SUN Project is a dynamic, prospective, multipurpose cohort of Spanish university graduates with a retention rate of 89%. We included in the analysis 9 365 participants free of overweight/obesity at baseline. At the baseline questionnaire, participants reported their postal code and the time they had been living in their city/village. We imputed the altitude of each postal code according to the data of the Spanish National Cartographic Institute and categorized participants in tertiles. We used Cox regression models to adjust for potential confounding variables.

**Results:**

During a median follow-up of 10 years, we identified 2 156 incident cases of overweight/obesity. After adjusting for sex, age, time of residence at current city, baseline body mass index, physical activity, sedentarism and years of education (≤ 3 years, ≥ 4 years, Master/PhD), those participants in the third tertile (>456 m) exhibited a statistically significant 14% reduction in the risk of developing overweight/obesity in comparison to those in the first tertile (<124 m) (adjusted HR = 0.86; 95% CI: 0.77, 0.96).

**Conclusions:**

Living in cities of higher altitude was inversely associated with the risk of developing overweight/obesity in a cohort of Spanish university graduates.

## Introduction

Obesity is an important public health problem worldwide. According to some recent estimates from the World Health Organization, more than 1.9 billion adults (39% of those 18 years or older) were overweight in 2014. Among them over 600 million had obesity (13%).

The consequences of these numbers on the population’s health are disturbing. Obesity is an independent risk factor for many Non-communicable diseases (NCD), responsible for 38 million (68%) of the world’s 56 million deaths in 2012 [1]. In Spain, a nationally representative survey carried out in 2011–2012 among individuals aged 18 years or older, showed that 53.7% were overweight and 17.0% were obese [2].

In the context of obesity development, environmental obesogenic factors [3,4] such as polychlorinated biphenyls (PCB) exposure either in adults [5] and children [6] have been reported because they can be modifiable. In this context of the environmental effects on weight gain, altitude has also been postulated as a potential factor to prevent obesity [7].

However, there are discrepancies in the results and it is not clear whether hypoxia triggers weight reduction or not. Studies have been conducted in a variety of time: short sojourns (days and weeks) [8] and long stays [9]; and in a variety of settings: moderate, high and extreme altitudes [10].

Beyond the multifactorial and complex development of obesity, at the end, this condition is the consequence of an imbalance between energy intake and energy output [11] accompanied with a mild chronic inflammation where one of the triggering factors, curiously, has been proposed to be hypoxia among others [12]. The relationship between the exposure to short, moderate and long term hypoxia conditions and body weight management has been previously investigated, but the mechanisms of hypoxia to induce body weight changes have not yet been fully understood. In higher altitudes there is an increase in basal metabolic rate (BMR), also an increase in leptin levels has been observed, leading to less energy intake due to lack of appetite. This results in a negative energy balance that may lead to weight loss [7,13]. However, there are inconsistent findings regarding leptin levels [8,14–17].

Given this hypothesis, some epidemiological studies have been conducted. Voss et al. [18] observed that obesity prevalence in the United States was inversely associated with elevation and urbanization. Also, in a quasi-experimental trial Voss et al. [9] found a lower obesity rate during residence at high altitude among a military population.

Because of the potential adaptive changes to altitude and the previous epidemiological results we hypothesize that there may be less risk of weight gain among those populations living at higher altitude. In addition, from a population perspective small differences in altitudes with modest adaptations may be more important than factors that have a clinical impact on a small number of people [19].

However, the association between long life exposure to unusual higher altitudes and the incidence of overweight/obesity has been scarcely assessed (altitudes defined in the literature are mostly: < 3.000/3.500 m moderate altitude, < 5.300 m high altitude, > 5.300/5.500 m extreme altitude) [20]. To our knowledge, this is the first study conducted in a free-living human population (in a not experimental setting) living in modest altitudes with the objective to assess prospectively the association between altitudes and annual weight gain and the incidence of overweight/obesity in a Spanish cohort (the SUN Project).

## Materials and Methods

### Study population

The “Seguimiento Universidad de Navarra” (SUN) project is a Spanish, dynamic, multi-purpose and prospective cohort study of university graduates. Initially it was designed to focus on diet and the occurrence of several diseases including overweight/obesity.

Information is updated biennially since December 1999. Details of its design have been published elsewhere [21]. Up to March 2012, the SUN project had recruited 21 291 participants. They were able to provide 2-year follow-up data by the end of 2014.

For the present analysis, we excluded those participants classified as overweight or obese (body mass index (BMI) ≥ 25 kg/m^2^) at baseline (n = 6340). We also excluded women who were either pregnant at baseline or became pregnant during the follow-up period (n = 3040) and participants who had been previously diagnosed with a chronic disease at baseline (including diabetes, cancer and cardiovascular disease (CVD); n = 1 061).

**Fig 1:**
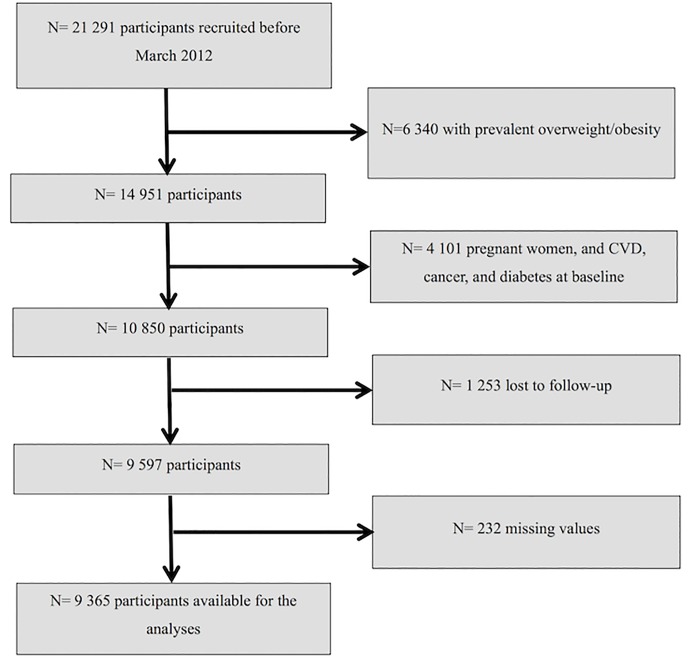
Flow-chart of participants included in the analyses.

Among the remaining participants, 1 253 were lost to follow-up leaving a total of 9 597 (retention rate = 89%). Additionally, we excluded 232 participants who had missing values in the variables of interest leaving a final sample of 9 365 participants available for the analysis (Fig 1).

The Institutional Review Board of the University of Navarra approved the study protocol. Voluntary completion of the first self-administrated questionnaire was considered to imply informed consent.

### Assessments of participants’ characteristics

The baseline questionnaire included questions about participants’ medical history, health-related habits, lifestyle and socio-demographic variables [21], as well as self-reported anthropometric data and the time of residence at the current city. Physical activity was ascertained through a baseline 17-item questionnaire.

The reproducibility and validity of self-reported anthropometric data [22], and physical activity [23] were assessed in subsamples of the cohort. A Mediterranean dietary pattern was defined “a priori” using the score (0–9 points) proposed by Trichopoulou et al. [24]

### Assessment of participants’ altitude of residence

The postal code of each participant’s residence and the time they have been living in their city/village were recorded at the baseline questionnaire. We imputed the altitude of each postal code according to the data of the Spanish National Geographic Institute using the program GIS ArcGIS v10.3 and categorized participants into tertiles. These tertiles ranged between: 0–122 meters (lower altitude), 123–456 meters (medium altitude) and 457–2 329 meters (higher altitude).

### Outcome ascertainment

Participants’ weight was recorded at baseline and on every follow-up questionnaire (every 2 years). BMI was calculated as the self-reported weight in kilograms divided by the square of height in meters.

Kappa index for self-reported vs. measured overweight/obesity was 0.91 (95% CI: 0.81, 0.99) in the anthropometrics validation study of a subsample of the cohort [21].

The outcomes were: 1) incidence of overweight/obesity (BMI ≥ 25 kg/m^2^); and 2) average yearly weight change (g/year).

### Statistical analyses

Baseline characteristics of participants according to their altitude of residence were described using relative frequencies, means and standard deviations.

Cox proportional hazards models were used to assess the association between the altitude of residence (roughly tertiles) and the risk of overweight/obesity during follow-up. Person-time of follow-up was calculated for each participant, from the date of completion of the baseline questionnaire until the date of completion of the last follow-up questionnaire, or date of death, whichever occurred first. For the cases of incident overweight/obesity, the person-time follow-up was set as the date of the follow-up questionnaire in which he/she was found to be overweight/obese.

Linear regression models were used to assess the association between the altitude of residence (roughly tertiles) and the average yearly weight change during follow-up, using the lower tertile of altitude of residence as the reference group. β-regression coefficients (and their 95% confidence interval (CI)) were estimated. The absolute yearly weight change in the 3 categories of altitude was calculated using the ANalysis Of VAriance test.

Tests of linear trend were conducted assigning medians of altitude for each category and treating this variable as a continuous variable.

Multiplicative interactions defined a priori between altitude of residence and age [older than 40 years], sex, total energy intake [low (under the median)/high (above the median)], weight change in the previous 5 years to entering the cohort [> = 3 kg of weight change] were tested using likelihood ratio tests comparing the fully adjusted model and the same model with the interaction product-term.

The Cox model included age as the underlying time scale for all analyses. We stratified all models by years living in the city/village, and categories of participants according to their date of entry in the cohort.

We fitted a multivariable model adjusted for age (deciles) and sex, and a multivariable model adjusted for more potential cofounders: baseline BMI (kg/m^2^), physical activity (MET-h/week in quartiles), sedentary behaviour (hours sitting down in quartiles), and years of education (≤ 3 years, ≥ 4 years, Master/PhD). A crude linear regression model was also calculated.

We conducted a Nelson Aalen plot to assess the incidence of overweight/obesity during follow-up according to the tertiles of altitude.

Sensitivity analyses were used to assess the robustness of our results under different scenarios. A relative index of sedentary lifestyle was calculated for each participant, assigning the 100% to the most sedentary participant and classifying the rest of the participants according to that value (sedentary lifestyle percentage).

In the same way, the most active participant (MET-h/week) was assigned 100% and the physical activity for the remaining participants was calculated as a proportion of this total (total activity percentage). We fitted a Cox regression model adjusted for quartiles of % sedentary behaviour (h/week)/% physical activity (METs-h/week) ratio, instead of adjusting for sedentary behaviour and physical activity.

Taking into account a different assumption of the induction period, Cox regression models were repeated using as outcome only those new cases of overweight/obesity occurring after 4 years of follow-up. We repeated the analysis after excluding participants with a personal history of obesity, familiar history of obesity and early incident cases of overweight/obesity (2 years of follow-up). We additionally adjusted in different sensitivity analyses for previous history of depression, and existence of respiratory diseases (asthma, emphysema, chronic bronchitis, and apnoea). To take into account those participants who moved during follow-up we repeated the analysis including only those who remained in their city/village.

Proportional-hazard assumption was tested with the Schoenfeld residual method. All p values presented are two-tailed; p < 0.05 was considered statistically significant. Analyses were performed using STATA/SE version 12.0 (StataCorp, College Station, TX, USA).

## Results

The main baseline characteristics of the participants according to their altitude of residence are described in Table 1. The mean age of participants was 37.01 ± 10.76 years (mean ± SD), and the mean BMI was 21.90 ± 1.92 kg/m^2^ (mean ± SD).

The higher category of altitude included participants with the least time of residence, and their BMI was the lowest. On average, participants in this category were less physically active and spent more hours sitting down. Also, participants who lived at higher altitude were less likely to follow a Mediterranean dietary pattern and a special diet, and reported snacking between meals more frequently.

**Table 1:** Characteristics of participants according to their altitude of residence. The SUN project 1999–2012.

	Altitude			
	Lower	Medium	Higher	
	0–122 meters	123–456 meters	457–2 329 meters	P value ^a^
Participants (n)	3 124	3 254	2 987	
Sex, n (% male)	1 127 (36.08)	1 029 (31.62)	1 005 (33.65)	0.040
Age (years), mean ± SD	38.14 ± 10.81	36.04 ± 10.55	36.89 ±10.84	<0.001
BMI (kg/m^2^), mean ± SD	21.99 ± 1.88	21.86 ± 1.93	21.85 ± 1.93	0.005
Total energy intake (kcal/d), mean ± SD	2536 ± 888	2594 ± 933	2588 ± 934	0.023
Mediterranean dietary pattern ^b^, mean ± SD	4.28 ± 1.80	4.18 ± 1.79	4.14 ± 1.79	0.007
Physical activity (METs-h/week), mean ± SD	24.30 ± 24.52	23.06 ± 24.65	22.03 ± 24.30	0.001
Sitting (h/d), mean ± SD	5.14 ± 2.09	5.17 ± 2.10	5.26 ± 2.04	0.086
Smoking status, n (%)				0.737
Current smoker	675 (21.61)	704 (21.63)	704 (23.57)	
Former smoker	812 (26.12)	784 (24.09)	732 (24.51)	
Incident CVD, diabetes or cancer, n (%)	234 (7.49)	192 (5.90)	222 (7.43)	0.899
Years of education, n (%)				0.001
≤ 3 years	857 (27.43)	1,050 (32.27)	902 (30.20)	
≥ 4 years	1,534 (49.10)	1,558 (47.88)	1,423 (47.64)	
Master/PhD	592 (18.95)	533 (16.38)	541 (18.11)	
Snacking between meals, n (%)	908 (29.07)	1,091 (33.53)	1,016 (34.01)	<0.001
Special diet followed, n (%)	205 (6.56)	195 (5.99)	152 (5.09)	0.015
Time of residence at current city (years), mean ± SD	21.09 ± 14.53	20.80 ± 13.40	19.90 ± 13.88	0.004

a. P value for comparison between-groups calculated by one-factor ANOVA for continuous variables or the χ2 test of linear trend for categorical variables.

b. Trichopoulou score (range of scores, 0 to 9, with higher scores indicating greater adherence).

Participants were followed-up for a median time of 10 years. During the follow-up period we observed 2 156 (23.02%) incident cases of overweight/obesity.

Participants in the highest category of altitude of residence lived at a median altitude of 583 m and according to their answer in the baseline questionnaire they were exposed at this altitude (they have been living in their city/village) for a median of 20 years. Those participants exhibited a significant inverse association with the risk of becoming overweight/obese during follow-up as compared to those in the lowest category (<123 m) of altitude of residence [HR = 0.86 (95% CI: 0.77, 0.96); p for trend = 0.006] (Table 2).

**Table 2:** Hazard ratios and 95% CI of incident overweight/obesity according to their altitude of residence. The SUN project 1999–2012.

	Altitude			
	Lower	Medium	Higher	
	0–122 meters	123–456 meters	457–2 329 meters	P for trend
Participants (n)	3 124	3 254	2 987	
Cases/person-years	763/23 821.63	736/25 742.39	657/24 492.67	
Multivariate adjusted ^a^	1 (Ref.)	0.94 (0.85, 1.05)	0.88 (0.79, 0.98)	0.018
Multivariate adjusted ^b^	1 (Ref.)	0.86 (0.77, 0.96)	0.86 (0.77, 0.96)	0.006

a. Adjusted for sex, age and time of residence at the current city.

b. Adjusted for sex, age, time of residence at the current city, BMI, physical activity, sedentary behavior and years of education.

Average yearly weight change as a continuous variable showed that participants who lived at >456 m gained less weight during follow-up than those who lived at <123 m (Table 3).

**Table 3:** Estimates (β-regression coefficients and 95% CIs) for participants’ average yearly weight change (g/year) according to their altitude of residence. The SUN project 1999–2012.

	Altitude			
	Lower	Medium	Higher	
	0–122 meters	123–456 meters	457–2 329 meters	P for trend
Participants (n)	3 124	3 254	2 987	
Absolute yearly weight change (g), mean ± SD	266.70 ± 679.99	240.02 ± 663.55	242.81 ± 657.96	
Crude	0 (Ref.)	-26.68 (-59.68, 6.31)	-23.90 (-57.45, 9.65)	0.137
Multivariate adjusted ^a^	0 (Ref.)	-34.56 (-67.64, -1.48)	-29.57 (-63.10, 3.95)	0.065
Multivariate adjusted ^b^	0 (Ref.)	-34.97 (-68.06, -1.88)	-33.55 (-67.04, -0.06)	0.038

a. Adjusted for sex, age and time of residence at current city.

b. Adjusted for sex, age, time of residence at current city, BMI, physical activity, sedentary behavior and years of education.

**Fig 2:**
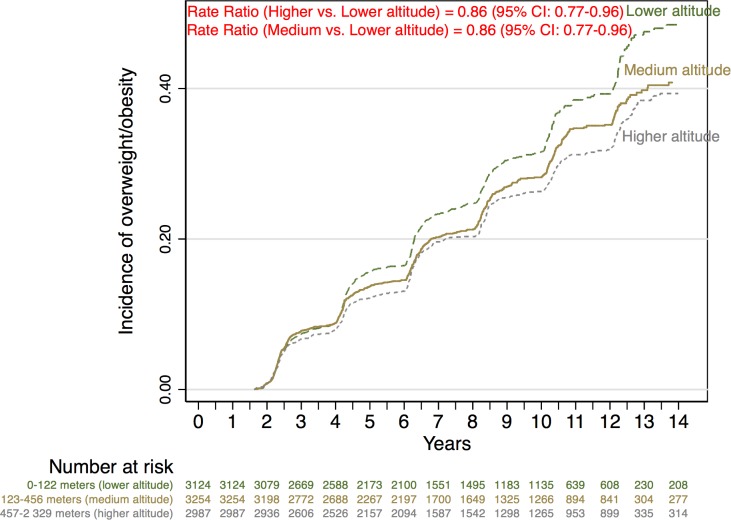
Nelson–Aalen cumulative hazard estimates.

The Nelson-Aalen survival function (Fig 2) shows the crude differences in the incidence of overweight according to tertiles of altitude of residence. These curves showed a lower incidence of overweight/obesity across increasing tertiles of altitude of residence.

We conducted an additional analysis. The highest tertile of altitude ranged from 457 to 2 329 m, which is a very broad range. Participants were divided into two extra categories.

However, there were only 21 participants who lived at > 1500 m. Both subgroups were associated with lower risk of becoming overweight/obese during follow-up as compared to those in the lowest category (<123 m): 456 to 1 500 m [HR = 0.86 (95% CI: 0.77, 0.97)], and 1 500 to 2 329 m [HR = 0.67 (95% CI: 0.25, 1.80); p for trend = 0.005].

No significant interactions were observed between altitude of residence and age [older than 40 years/younger than 40 years; p for interaction = 0.648], sex (p for interaction = 0.954), total energy intake [low (under the median)/high (above the median); p for interaction = 0.081], weight change in the previous 5 years to entering the cohort [> = 3 kg of weight change; p for interaction = 0.884].

We performed multiple sensitivity analyses. In all these sensitivity analyses the point estimates were in the same direction as the estimates observed in the main analysis, although after excluding participants with familiar history of obesity results were not statistically significant (Table 4).

**Table 4:** Sensitivity analyses. Hazard ratios and 95% CI of incident overweight/obesity in participants according to their altitude of residence. The SUN project 1999–2012.

		Altitude			
		Lower	Medium	Higher	
	Cases/person-years	0–122 meters	123–456 meters	457–2 329 meters	P for trend
Overall	2 156/74 056.69	1 (Ref.)	0.86 (0.77, 0.96)	0.86 (0.77, 0.96)	0.006
Excluding participants with depression	2 061/71 134.48	1 (Ref.)	0.85 (0.76, 0.95)	0.86 (0.77, 0.97)	0.007
Excluding participants with respiratory disease	1 980/68 658.56	1 (Ref.)	0.88 (0.78, 0.97)	0.87 (0.78, 0.98)	0.012
Excluding participants with personal history of obesity	2 068/72 915.61	1 (Ref.)	0.87 (0.77, 0.97)	0.86 (0.77, 0.96)	0.006
Excluding participants with family history of obesity	1 631/59 654.69	1 (Ref.)	0.89 (0.78, 1.01)	0.89 (0.78, 1.01)	0.061
Excluding early incident cases of overweight/obesity (until 2 years of follow-up)	1 443/72 297.94	1 (Ref.)	0.79 (0.69, 0.90)	0.82 (0.72, 0.94)	0.002
Cox regression at 4 years of follow-up	1 171/76 688.50	1 (Ref.)	0.89 (0.77, 1.02)	0.83 (0.71, 0.97)	0.013
Cox regression adjusted for the ratio % sedentary behaviour (h/week)/% physical activity (METs- h/week)	2 156/74 056.69	1 (Ref.)	0.86 (0.77, 0.96)	0.85 (0.76, 0.95)	0.003
Excluding participants who moved during follow-up	1 655/57 578.17	1 (Ref.)	0.86 (0.76, 0.98)	0.89 (0.78, 1.01)	0.041

A non-significant result for the Schoenfeld residual method was found (p = 0.702) suggesting no evidence of a departure from proportionality of hazards.

## Discussion

In this large prospective cohort we observed that participants living at higher altitude had a modest, but significantly inversely associated, average yearly weight gain and a lower risk of developing overweight/obesity. These associations were assessed independently of a variety of potential cofounders, which were previously described.

At sea level, atmospheric O_2_ partial pressure (pO_2_) is approximately 160 mmHg and decreases proportionally as the barometric pressure decreases (1 mmHg each 10 m up). In our dataset participants in the highest category of altitude lived at a median 583m and they were exposed, for a median time of 20 years, to an standard barometric pressure of 95kPa, in other words to 94% of the oxygen available at the sea level. This oxygen pressure translated to the body’s oxygen supply taking 12 breaths per minute as the respiratory frequency and a tidal volume of 0.65 liters means an arterial pO_2_ of 11kPa [25].

By contrast, the median altitude of those who were living in the lowest altitude (45m) had an atmospheric pO_2_ of 101kPa (with almost 100% of oxygen available at sea level). Using the same previous conditions this atmospheric pO_2_ means an arterial pO_2_ of 12.2kPa.

This difference of 1.2kPa is not large, and these calculations have their own limitations in this context, since they do not take into account adaptations over years and generations. However, although the difference is small, the existence of potential adaptive mechanisms for long-time exposure to this level of hypoxia may exist. In the field of sports, exercise-induced arterial hypoxaemia is defined as a reduction in the arterial oxygen pressure only higher than 1kPa [26].

This leads to various physiological mechanisms following adaptation due to chronic lower hypoxia. In addition, from a population perspective we have to take into account the potential existence of Rose prevention paradox where factors that make a small difference to the population distribution may be more important than factors that have a clinical impact on a small number of people [19].

Previous studies have assessed the role of hypoxia on body weight management. A study conducted in 20 obese males found that, after a 1-week stay at high altitude (2650 m), BMR was higher and food intake and diastolic blood pressure were significantly lower. Also, leptin levels were significantly increased despite the reduction in body weight [8].

However, some studies have found a reduction of leptin levels at high altitude [17]. And Woolcott et al. [15] reported no statistical difference between plasma leptin levels in two different populations of dwellers from Andes. It remains a controversial subject and other hormones, like Ghrelin, may be also one of the candidates that lead to lack of appetite [27]. Vats et al. [28] observed that on altitude exposure there was a significant decrease in body weight, levels of total body water, extra cellular and intra cellular body water.

Other plausible biological mechanisms leading to weight loss at higher altitudes have been hypothesized: increased resting metabolic rate, the role of molecules like NPY, ghrelin, galanin, CCK and IL-6 [29–33]. However, the negative energy balance in hypoxia seems to be largely due to a reduction in energy intake from a lack of appetite [12]. Eating less will lead to body size reduction and subsequently to being able to survive with a smaller oxygen consumption.

Epidemiological studies on the subject are scarce but may shed some light on the mechanisms related with higher altitude adaptation. Consistent with our findings, Voss et al. [18] observed in a cross-sectional study of 422 603 US adults that after controlling for urbanization, temperature category and behavioral and demographic factors, male and female Americans living <500 m above sea level had 5.1 (95% CI: 2.7, 9.5) and 3.9 (95% CI: 1.6, 9.3) times the odds of obesity, respectively, as compared with counterparts living >3000 m above sea level.

Furthermore, in a quasi-experimental study conducted among a military population, service members stationed at high altitude had a lower hazard ratio of incident obesity compared to those at low altitude (HR = 0.59 (95% CI: 0.54, 0.65)) [9]. In addition, Woolcott et al. [34] could demonstrate that US individuals living at high altitude had 25% lower odds of being obese than those living below 499m.

Some studies of native populations living at high altitude have also been conducted but most of them with important limitations such as the use of a cross-sectional design [10,35].

The results of our study open some questions about the potential utility of hypoxia against overweight/obesity. However, few studies have aimed to assess the potential benefits of hypoxia as a treatment for obese people.

In a blinded randomized trial Netzer et al. [36] recruited 32 obese people for a mild intense training in normobaric hypoxia (15% O_2_) and normoxia/sham hypoxia (21% O_2_) for 8 weeks. Results indicated that low intensity exercise training in normobaric hypoxia may lead to more weight loss than such training in normoxia (-1.14 vs. -0.03 kg). Interestingly, the same authors found no effect on weight and other parameters on obese subjects after exposing them to normobaric intermittent hypoxia for 8 months [37].

Furthermore, two other studies published contradictory results, while in the article from Kong et al. [38], young overweight adults lose more weight training under normobaric hypoxia than in normoxia after 4 weeks in a residential camp; in the article from González-Muniesa et al. [39], obese patients suffering sleep apnea-hypopnea syndrome did not increase their weight loss due to a hypoxic environment. All these studies have been designed simulating altitude hypoxia with different equipment. These results strongly suggest that there might be a factor or factors (such as the aforementioned and probably others) occurring in real altitude that have not been reproduced properly with these instruments.

Our study has several potential limitations and we should be very cautious about the causality of our results. First, biological plausibility is scarce at the moderate altitudes we studied and extensive research is still warranted to clarify the mechanisms that support our hypothesis.

In addition, the between-group difference in our continuous outcome (annual change in body weight by 35g) was not clinically meaningful. Nevertheless, this observation supported our findings on the incidence of overweight/obesity, which is indeed a clinically relevant outcome. In addition, small average weight gains every year during follow-up if they are persistent in the long-term might produce a life-long increased incidence of overweight/obesity at the population level.

Our outcome was based on self-reported data. Therefore, inherent to our methodology we have a potential information bias. Nevertheless, a similar degree of misclassification between exposure categories would be expected and probably the estimates were expected to be closer to the null value. Furthermore, self-reported weight and BMI were previously validated in a subsample of the cohort finding good validation results [22].

Our cohort is restricted to university graduates, therefore we should be cautious about the generalizability of our results. Since our study was conducted in Spain, there is only a limited variability in our exposure variable; participants living at even higher altitudes would have been preferred.

We should take into consideration the possibility of selection bias due to our sample restricted to university graduates. However, it is difficult to conceptualize that having a higher education is related to living at lower or higher altitudes. For example, the two main Spanish cities, Madrid and Barcelona, are at different altitudes. Madrid is at 667 m and Barcelona is at sea level. Nonetheless, in the case of this selection bias, graduates supposedly with less risk of obesity may live at lower altitudes than other type of population, such as farmers and ranchers. Thus, taking into account that education is a proxy of socioeconomic status (SES) [40] and lower socioeconomic status is related to higher risk of overweight/obesity, this potential selection bias would cause an underestimation of the observed effect.

Unfortunately, we do not have information about SES of our participants. However, although educational level does not warrant a similar income, as we have mentioned before, it can be considered a proxy of SES [40]. Education was the strongest determinant of socioeconomic differences in analyses that have taken into account education, occupation, income, and employment status [41]. All our participants were university graduates. Therefore, we are confident that SES cannot be a major confounder (ie. we used restriction to control for SES). In addition, we have adjusted the analyses for years of education of our participants.

Some degree of residual and time-depending confounding cannot be discarded despite we performed multivariable analyses adjusting for multiple potential confounders, for example, lifestyle itself which carries further characteristics beyond e.g. BMI, physical activity, age, etc. Naturally, comprehensive lifestyle is hard (or impossible) to measure. To avoid falling into an ecological interpretation of our data and a potential selection bias (perhaps selected people live/move/remain living at higher altitudes), we assessed the time of residence at current city but the self-reported nature of the variable is a potential limitation. However, self-reported weight and BMI were previously validated in our cohort [22].

Our results are statistically significant, despite the proximity to the upper limit of the CI to the null value. In addition, we cannot rule out the existence of type I error or residual confounding.

The strengths of our study include its prospective design with a long follow-up and a relatively large sample size with high retention rate. We count with a sufficient number of overweight/obesity cases and detailed and repeated assessments of lifestyle variables over the course of the study.

## Conclusions

In conclusion, this study showed that living in cities of higher altitude was inversely associated with the risk of developing overweight/obesity and weight gain in a cohort of Spanish university graduates. However, future studies in higher altitudes are still needed and extensive research must be conducted to prove the role of hypoxia by living at modest higher altitudes as a causal element for weight loss.
